# Three dimensional printing of metamaterial embedded geometrical optics (MEGO)

**DOI:** 10.1038/s41378-019-0053-6

**Published:** 2019-04-08

**Authors:** Aydin Sadeqi, Hojatollah Rezaei Nejad, Rachel E. Owyeung, Sameer Sonkusale

**Affiliations:** 1 Nano Lab, Department of Electrical and Computer Engineering, Tufts University, Medford, MA 02155 USA; 2 Department of Chemical and Biological Engineering, Tufts University, Medford, MA 02155 USA

**Keywords:** Microwave photonics, Micro-optics

## Abstract

Three-dimensional printers have revolutionized many scientific fields with its low-cost, accessibility and ease of printing. In this paper, we show how stereolithography (SLA) based 3D printers can enable realization of innovative 3D optical devices formed through the fusion of metamaterials with geometrical optics or MEGO. It utilizes a combination of desktop SLA 3D printer and metal deposition/coating systems. Using this approach, we present innovative metamaterial embedded optical components such as mushroom-type metamaterials, curved wide-angle metamaterial absorbers/reflectors and a frequency selective moth eye hemispherical absorber. Finally a unique MEGO device formed through the fusion of a frequency selective metamaterial with an optical parabolic reflector has been demonstrated that combines their individual properties in a single device. The fabricated MEGO devices operate in the millimeter wave frequency range. Simulation and measurement results using terahertz continuous-wave spectrometer validate their functionality and performance. With improving resolution in 3D printing, MEGO devices will be able to reach Terahertz and optical frequencies in the near future.

## Metamaterials: multi-functional optics emerge from 3D printers

A technique that uses large area 3D printing and metal layer patterning can realize complex optical devices at a fraction of the cost of typical fabrications. Sameer Sonkusale and colleagues from Tufts University in the United States designed geometric surface patterns, such as tiny forests of mushroom-topped cylinders, capable of interacting strongly with microwave light. Optimizing the conditions needed to 3D print these arrays using photo-curable polymers and to coat them with reflective gold or silver layers led the researchers to explore other unique device possibilities. They created a surface capable of selective high-frequency energy absorption by patterning hemispherical surfaces that resemble an insect’s eye. Combining the frequency selective metamaterials with a 3D-printed parabolic lens yielded a prototype that could reduce the expense and size of conventional terahertz spectrometers.

## Introduction

3D printing is an additive manufacturing technique for fabricating structures and devices with different geometries using computer-aided design. The process includes printing successive layers of a given material on top of each other^1^. There are primarily four approaches to additive manufacturing, fused deposition molding (FDM), selective laser sintering (SLS), inkjet printing and stereolithography (SLA). In FDM method, a filament of thermoplastic polymer is heated at the nozzle to reach a semi-liquid state and then extruded on the platform. There has been growing trend to make conductive filaments for FDM based 3D printers making them suitable for electronics and electromagnetic applications^2^. SLS process uses targeted laser beam to melt and fuse powders in a powder bed to form 3D structures. Inkjet printing has also been used for additive manufacturing of ceramics. It is used for printing complex and advanced ceramic structures for applications such as scaffolds for tissue engineering. SLA is another approach for 3D printing which uses focused light to polymerize photo-curable resins. Using a movable stage, one can cure resin to form 3D structures (e.g., Formlabs^3^ printer). Some other printers (e.g., Photonic Professional GT by NanoScribe^4^) even offer resolution down to 200 nanometers using two photon polymerization (TPP)^5^. TPP technique provides high resolution but is very low throughput method for 3D printing. Furure TPP printers may have better throughput. All the 3D printing technologies mentioned above have revolutionized many scientific fields due to the ability to prototype designs rapidly. For example, they have been used to make prosthetic limbs^6–11^, dental crowns^12^, organs-on-a-chip^13^, microneedles^14–16^ and wearables^17^. 3D printers have also been used in electronic, optical and photonic applications such as metamaterials^2,18–21^ which is also the focus of this paper.

Metamaterials (introduced by Victor Veselago in 1968)^22^ are artificially engineered materials, which can be designed to show unique electromagnetic properties sometimes not found in nature. They can be designed to exhibit effective negative permittivity or permeability, epsilon-near-zero or mu-near-zero behaviors for variety of applications such as absorbers, phase shifters, modulators, sensors, etc^23–38^. Exciting developments in metamaterials were ushered in with access to 3D printers with nanoscale features. They were used to print chiral metamaterials, photonic crystals, tunable plasmonic surface and optically actuated surface scanning probe and circular polarizers at optical frequencies^39–45^. Electroplating has shown a good compatibility for making a conductive layer on devices with very small feature size fabricated by TPP method^45^. However, those devices are usually small of the order of 500 × 500 μm^2^ area. In spite of this early promise, we believe the true potential of 3D printers has not been fully realized.

In this paper we propose a hybrid fabrication approach including 3D printing, metal coating and wet etching to realize 2D and 3D metamaterials with complex geometries and novel functionalities. One contribution is using this approach to fabricate angle insensitive metamaterials that conventionally require multiple steps of photolithography on a curved substrate^45^ or requires metamaterials to be printed on flexible substrate^36,46–51^ which is then draped over a desired 3D printed device. However only limited 3D metamaterial designs can be implemented using this approach. On the other hand, the proposed method enables three dimensional pattering of dielectric layers which when combined with the ability to pattern metal layers can provide access to unique electromagnetic functionality. For example, we made mushroom like metamaterials to operate as Gigahertz absorbers. Another contribution is the ability to fuse multiple electromagnetic functions, which traditionally are achieved by using different optical components, into a single metamaterial embedded geometrical optics or MEGO device. For example, we consolidated the optical parabolic reflectors with frequency selective transmissive filters operating around 100 GHz into a single MEGO frequency selective parabolic mirror. This MEGO device effectively realizes a frequency selective focusing lens. One could also take advantage of the large area 3D printing of dielectrics with the embedding of metal patterns. For example, we show a new MEGO device namely an omni-directional hemispherical moth-eye lens except it is made frequency selective for angle-insensitive omnidirectional absorption and detection.

## Results

### Design and fabrication of mushroom MEGO

**Fig. 1:**
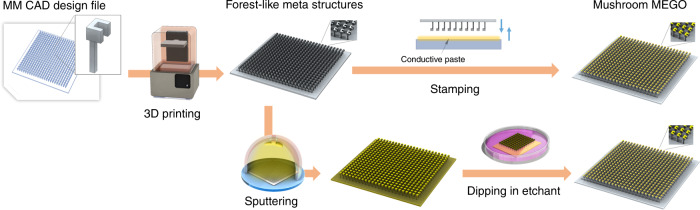
Schematic view of fabrication flow. We design the model in 3D CAD software. Then we print the model by 3D printer. In our first approach (first row in the figure) we coat the top surfaces of mushroom MEGO with conductive paste (stamping method). In our second approach (second row in the figure) we sputter metal on the whole 3D printed device and then submerge the device in etchant to etch away the existing metal on the pedestal and the substrate

We showcase the promise of proposed fabrication approach that combines SLA based 3D printing, metal coating and wet etching to design, implement and validate several MEGO devices. As a first example, we explore Mushroom-type metamaterials on pedestal (Mushroom MEGO). Mushroom-type metamaterials^52–56^ are structures that resemble a long pedestal holding a patterned metal resonator on top. Mushroom MEGO enables design of absorbers or reflectors over a wide range of frequencies by controlling both the resonator geometry and the pedestal itself. However these metamaterials are challenging to fabricate by conventional photolithography since the dielectric medium underneath the metamaterial needs to be shaped in a three dimensional pattern. The fabrication process is shown conceptually in Fig. 1. We input the design of array of split ring resonators (SRR) or disk-shaped resonators on a pedestal as our metamaterial. This design is then transferred to the SLA-based 3D printer for printing. We used high temperature resin for our fabrication, provided by Formlabs. The resin had permittivity of ε (100 GHz) = 2.6 + j 0.0837 and *ε* (200 GHz) = 2.5 + j 0.05718 having low loss for our purpose. The resulting device is then washed with Isopropyl alcohol (IPA from Sigma-Aldrich, Natick, USA) and water respectively. Finally metal is deposited on the top metamaterial surface. Note that metamaterial resonator is realized with desired geometry on the underlying 3D printed structure without the need for photomask and conventional photolithography. In our first approach for metal coating, we dip the metamaterial to stamp silver paste on 3D printed design to realize the resonators. In our second approach, we sputter metal (gold) layer on the whole device, note that other deposition methods like chemical vapor deposition or atomic layer deposition can be used instead of sputtering. We then remove gold from unwanted areas between the resonators and from the substrate by wet chemical etching. For this, we dip the device in a gold etchant to etch away the coated regions except for resonators’ top surface, then we rinse the device with distilled water. Since stamping is performed manually there is more variability in the thickness and uniformity of metal coating compared to the sputtering approach.

**Fig. 2:**
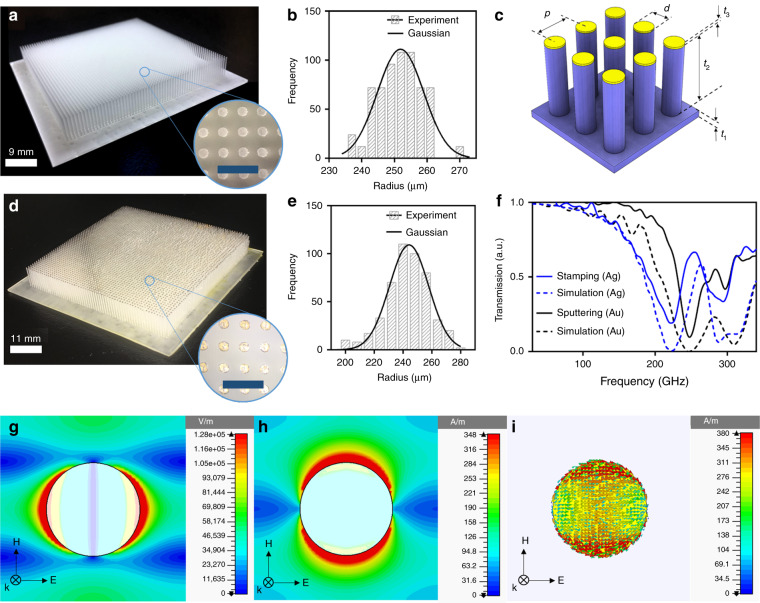
Cylindrical mushroom MEGO **a** cylindrical pillar arrays before coating and focused view of pillar metamaterial, the scale bar is 2 mm for magnified picture **b** the variability of the effective radius of the dots before coating **c** schematic figure of the device with t_1_ = 1 mm, t_2_ = 8 mm, t_3_ = 100 μm/100 nm (stamping/sputtering), d = 0.5 mm and p = 1 mm. **d** Pillar metamaterials after coating with silver and focused view of pillar metamaterial, the scale bar is 2 mm for magnified picture **e** the variability of the effective radius of the dots after stamping **f** transmission spectrum of the device by stamping and sputtering approaches comparing to the theoretical result **g** electric field distribution **h** magnetic field distribution **i** surface current density

As our first mushroom type MEGO device, we designed simple cylindrical pedestals on a substrate which makes it look like a forest structure as shown in Fig. 2a. The 3D designs are done in SolidWorks (Dassault Systèmes SolidWorks Corporation; Waltham, MA, USA)^57^. The structures were printed using a commercial Form 2 printer supplied by Formlabs Incorporated (Somerville, MA, USA). After printing our design with high temperature resin and printing resolution of 25 μm as shown in Fig. 2a, we characterized the surface area of disks and plotted the variability of the disks shown in Fig. 2b. We design cylindrical pedestals with a radius of 250 μm and unit cell of 1 mm but as we expect, we see variability in the surface area of the disks ranging from 235 μm to 270 μm showing a Gaussian distribution centered at 250 μm. After printing out the structure, we coat the top surface of the cylinders with silver paste (AG-510 Silver conductive ink, Applied Ink Solutions; Westborough, MA, USA) by stamping method (Fig. 2d). For our second fabrication approach, we sputter 100 nm of gold on the 3D-printed device using NSC-3000 Magnetron sputter tool and then we etch away the gold from the whole device except the top surface using gold etchant type TFA (supplied by Transene Company, Incorporated; Danvers, MA, USA). To have a better etching outcome, we plasma etch the device for 1 min before starting the gold etching process. Plasma etching enables the etchant to flow easily on the substrate leading to cleaner removal of metal from the underlying layer. The disk-shape mushroom MEGOs are schematically shown in Fig. 2c showing all critical dimensions; it has a diameter of 500 μm with pedestal height of 8 mm. Note that there is no restriction on achievable aspect ratio and one can choose any height for the pedestals. It was arbitarily chosen to be 8 mm, high enough to have an easier task of etching. The substrate thickness is 1 mm. Resonators are spaced 1 mm apart and the thickness of coated gold metal is 100 nm in sputtering approach and an average of 100 μm of silver in the stamping method. Stamping method has low resolution and there is not much control on thickness of the inked layer. We took an image and measured the thickness of the stamped silver for different unit cells using ImageJ and used this to report an average height of 100 μm for silver using this method. The wet etching process of sputtered disk resonators is shown in Supplement 1, Fig. S1. Since the stamping method is a non-uniform coating method, we characterized the surface area of the coated disk-shaped resonator to compare the surface area before and after coating. The coated areas by stamping method show high variability in the coated surface area (see Fig. 2e) but it remains a Gaussian distribution. The number of silver coated disk resonators with lower radius than 250 μm has increased due to the quality of stamping method. Since some disk surfaces are not coated thoroughly, then the coated area is smaller than the actual disk surface. The transmission spectrum was extracted for samples coated by sputtering and stamping approaches showing a resonant frequency at 248 GHz for gold sputtered metamaterial device and 222 GHz for silver stamped one, and it is compared with simulation results in Fig. 2f. The second resonance at 300 GHz is caused by higher order effect notably interference between unit cells. For experimental validation, we use a continuous-wave terahertz spectrometer^58^ with setting specifications discussed in details in references ^24,33,59^. The electric field distribution, magnetic field distribution and surface current density in simulation are shown in Fig. 2g–i. The results show that conventional 3D printing using commercial resins enable high frequency operation. For higher frequency applications the surface roughness will affect the performance of the device, the device would need smoothening of the surface by Polyurethane coating discussed in details for a parabolic MEGO reflector. Surface roughness of the device is shown with an atomic force microscope (AFM) image in Fig. S2 of the supplementary section.

**Fig. 3:**
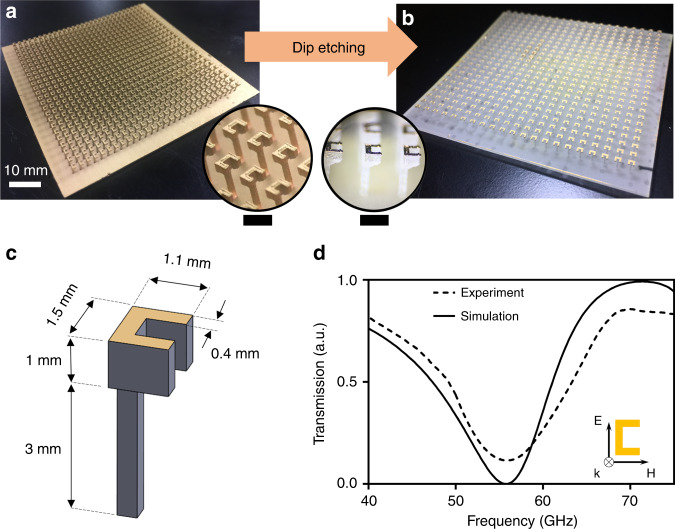
Split ring resonator (SRR) Mushroom MEGO **a** Gold sputtering on 3D printed structure followed (Scale bar 2.5 mm) by **b** etching underneath to realize MEGO (scale bar 2 mm) **c** feature sizes of the fabricated SRR Mushroom MEGO with unit cell size of 2.5 mm **d** transmission spectrum of the SRR Mushroom MEGO showing resonant frequency at 55.87 GHz

We also extended our fabrication method for different resonator structure to show its versatility. The split ring resonator is shown in Fig. 3a, b before and after etching. The dimensions of the split ring resonator are displayed in Fig. 3c. The transmission spectrum is displayed and compared with simulation result in Fig. 3d, the SRR is showing a resonant frequency at 55.87 GHz. Fabrication steps of SRR Mushroom MEGO is shown in Supplement 1, Fig. S3.

**Fig. 4:**
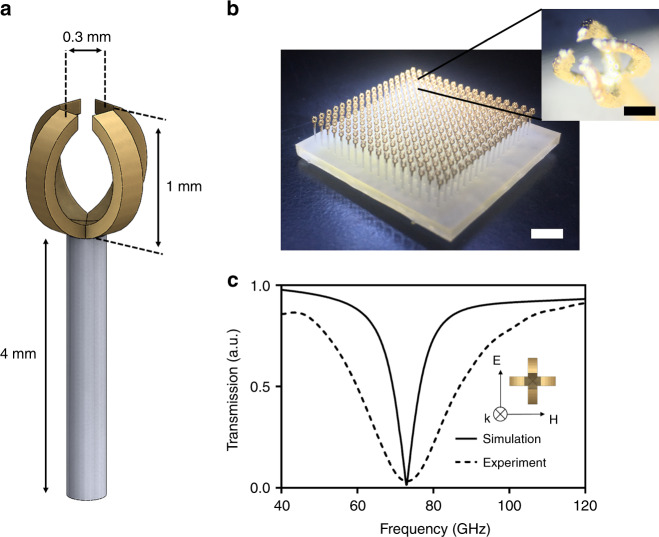
Two split ring resonator with 90 degrees of circular rotation Mushroom MEGO **a** the dimensions are shown schematically **b** the device with scale bar of 2 mm and a magnified image showing the split ring with scale bar of 0.3 mm **c** transmission spectrum with resonant frequency of 73 GHz

So far, we have shown disk and split ring resonators which are planar and can be fabricated by photolithography but there are some truly three dimensional resonators that cannot be fabricated by conventional photolithography or may require multiple steps (>10) of photolithography. Even injection molding may not be possible due to discontinuities, sharp curvatures and higher aspect ratios in some 3D designs^60^. In Fig. 4a we show two split ring resonators with 90 degrees of circular rotation with respect to each other, the radius of each ring is 500 μm, the gap of each SRR is 300 μm and the unit cell size is 1.25 mm. Figure 4b shows the final device with magnified image of a unit cell. Transmission spectrum is shown in Fig. 4c, displaying resonant frequency at 73 GHz.

### Novel MEGO components

The approach presented in this paper provides opportunities to create novel metamaterial embedded geometrical optics elements. We present two such novel designs. First we show an omni-directional hemispherical moth-eye absorber and show its insensitivity to propagation vector. Secondly we show the fusion of a frequency selective metamaterial with an optical parabolic reflector.

### Omni-directional hemispherical moth-eye MEGO absorber

**Fig. 5:**
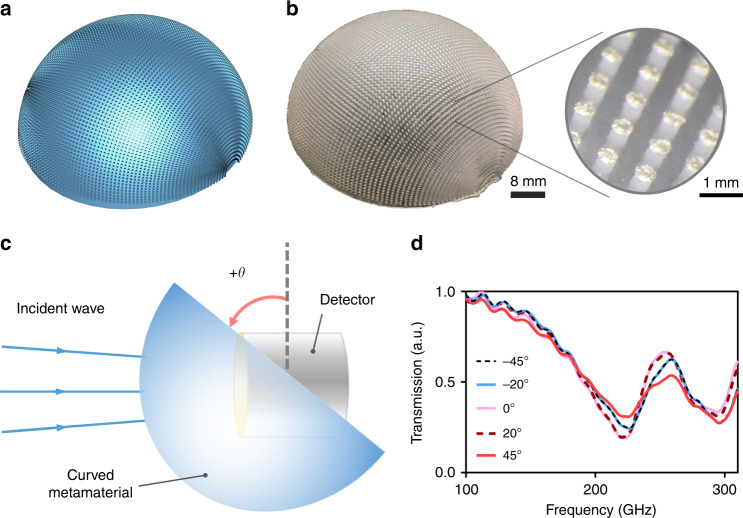
**a** Computer-aided design of the hemispherical moth-eye MEGO absorber **b** 3D printed and silver coated moth-eye MEGO absorber with magnified image **c** schematic of the device in different propagation angles as a function of θ **d** transmission spectrum of the omni-directional hemispherical moth-eye MEGO absorber as a function of θ

We took the planar mushroom MEGO design from previous section and made it in the mold of a hemisphere resembling a moth eye calling it a moth-eye absorber as seen in Fig. 5a schematically. We 3D print the design by SLA-based printer. The 3D printed device is shown in Fig. 5b (The edges are hard for the printer to print however these edge artifacts do not affect the overall response of the metamaterial), then we coat the top surfaces with silver paste by stamping method. The printed moth-eye absorber with silver coatings is shown in Fig. 5b with a magnified image of the silver-coated resonators. We measure transmission spectrum using continuous-wave Terahertz spectrometer for different angles of incidence. The rotational movement of the device is shown in Fig. 5c schematically, and the transmission spectrum with different propagation angle is shown in Fig. 5d. The results show identical transmission spectrum for angles of incidence from −45° till +45° with only slight minor differences. These differences can be attributed to the non-uniformity in stamping. We also show the different angle of incidence schematically in Fig. S4 in the supplementary section. Sputtering and wet etching approach is not suitable for the fabrication of this device since the etchant cannot overflow easily beneath the disk resonators due to the curvature of the substrate. To the best of our knowledge, this is the first ever realization of an angle‐insensitive narrow‐band metamaterial absorber in the form of hemispherical moth-eye absorber fabricated on curved substrate We believe such moth-eye absorbers can be quite useful. For example, it will improve the responsivity of photo detectors by absorbing all the incident electromagnetic radiation. It can also be used to make the next generation of cloaking devices. While only single frequency structure was shown, one can print different resonators to achieve multi-band or even broadband spectrum in a moth-eye MEGO absorber.

### Parabolic MEGO reflector

To consolidate an optical parabolic reflector with a frequency selective transmissive filter, we can also consolidate different optical and metamaterial functionalities into a single MEGO device. For example, we can consolidate an optical parabolic reflector with a frequency selective metamaterial based transmissive filter to realize a unique parabolic MEGO reflector device. The fabrication process is similar to the approaches described earlier and involves 3D printing followed by metal coating and etching with some modifications. We first design a parabolic substrate with the same parabola dimensions as 90° off-axis parabolic mirror (MPD762762-90-M01 by Thorlabs; Newton, NJ, USA), the dimensions of our parabolic reflector is shown in Supplementary, Fig. S5. After printing out the parabolic substrate, the device is post-cured in Form Cure (by Formlabs) in 60 °C for an hour. Post-curing hardens the device and there is less deviation from initial specifications. The printed and post-cured substrate surface is not smooth but is highly diffusive. Therefore we sand polish the surface of the substrate with sandpapers of grits 120–3000 (Miady; China) and sandpapers of grits 5000 and 7000 (Starcke®; Germany). After sanding the surface with high grit sandpapers, we rinse the surface with acetone, IPA and distilled water respectively and remove all particles with blowing nitrogen gun on the surface. Next, we pour fast drying Polyurethane (Minwax®; Upper Saddle River, NJ, USA) on the surface and let it dry out for 2 hours. Then we deposit 20 nm of Chromium and 200 nm of gold as the ground plane of the device.

**Fig. 6:**
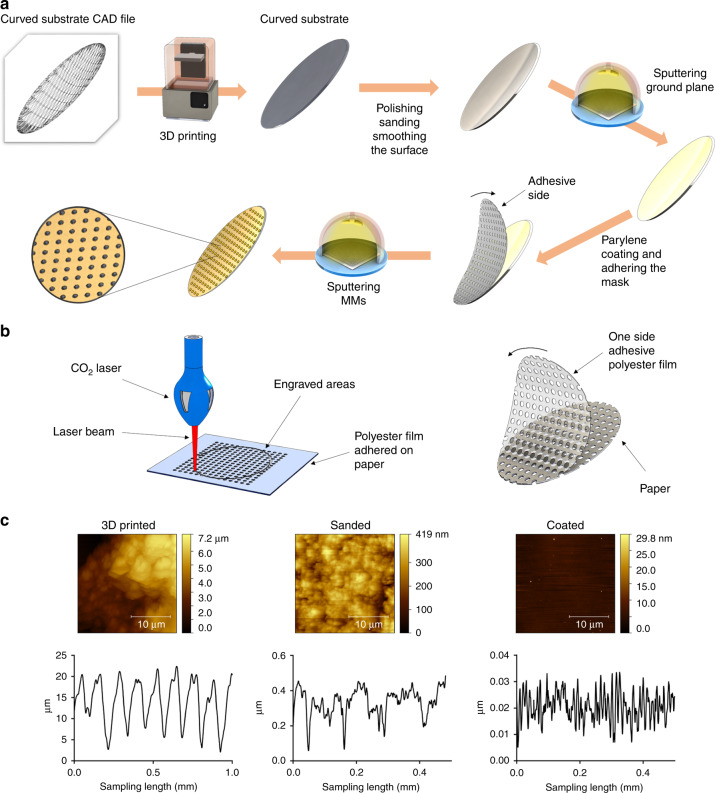
**a** Fabrication flow of parabolic MEGO reflector **b** making the adhesive mask by engraving a PET sheet with CO_2_ laser **c** atomic force microscopy images of the parabolic MEGO reflector surface in an area of 25 × 25 μm^2^ followed by surface roughness measured with dektak-XT Stylus for 3D printed, sanded and coated surface, respectively

After gold deposition, we deposit 60 μm of Parylene-C as the dielectric layer (PDS2010 Parylene coater from Specialty Coating Systems^TM^; Indianapolis, IN, USA). We designed a low-cost stencil mask for patterning metamaterials using AutoCAD^61^ by Autodesk (San Rafael, CA, USA). We laser-cut (Boss LS-1416, SKU: LS1416DX, Sanford, FL, USA) the Polyester film with adhesive back (part number 8689K42 from McMaster-Carr Robbinsville, NJ, USA) with this metamaterial pattern. For this proof of concept, we chose a simple circular disk resonators as metamaterial patterns with the radius of 500 μm and unit cell of 2 mm. The laser cutter is set to 10 mm/s of speed and 9 W of power. Then we place the printed mask on the parylene layer and sputter 20 nm of chromium and 200 nm of gold, after sputtering we put the device in acetone beaker so that Polyester film would peel off easily without damaging the parylene layer. Consequently we rinse the surface with IPA and distilled water. This fabrication procedure for MEGO device is shown in Fig. 6a. The fabrication of the mask by CO_2_ laser engraving is shown in Fig. 6b. As evident from the proposed approach, we trade-off a few additional manual steps after the 3D fabrication (coating and polishing) to enable realization of high performance optical devices such as curved mirrors at fraction of the overall cost of commercial mirrors. Beyond mirrors, the proposed approach enables realization of complex high frequency high performance metamaterial embedded optical devices. From AFM images shown in the Fig. 6c, there is a clear difference in surface roughness with each processing step, roughly an order of magnitude difference for the 3D printed sample, sanded, and coated samples shown in the figure. Images were taken on a Flex-Axiom AFM and c3000 controller (Nanosurf AG, Liestal, Switzerland) with an ACLA-10 silicon tip (Applied NanoStructures, Inc. Mountain View, CA, USA) in dynamic mode. Images were created using Gwyddion Software (Czech Metrology Institute). We also measured the roughness of the surface for parabolic reflector’s surface with Dektak-XT Stylus by Bruker, Tucson, Arizona, USA. Surface roughness was measured for the device after printing, sanding and coating with Polyurethane. This technique covers a much larger area versus AFM which could account for differences in the magnitude. Regardless, both techniques show approximately an order of magnitude difference between printing, sanding, and coating, respectively. As displayed in the Fig. 6c the roughness of the surface is decreased drastically through in each step. After finishing the coating it is seen that the roughness of the surface in the last step after coating is about 10–20 nm.

**Fig. 7:**
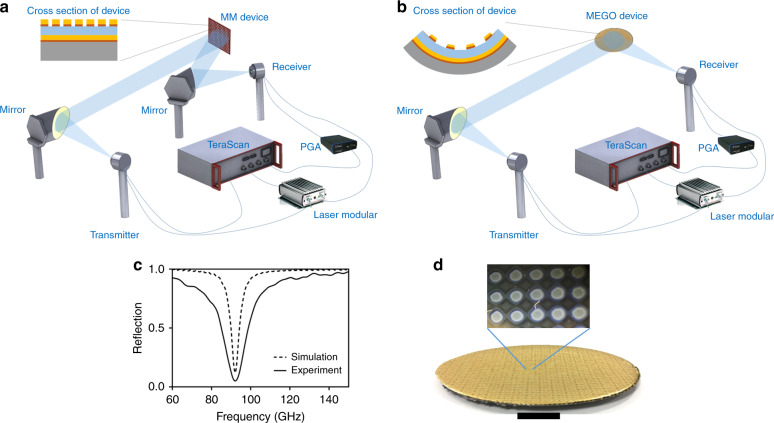
**a** Continuous wave Terahertz spectrometer setup for conventional reflection measurement with regular metamaterial device **b** reflection measurement with parabolic MEGO reflector **c** Reflection spectrum of the MEGO device **d** the fabricated metamaterial on parabolic surface (scale bar is 3 cm) with magnified image, each dot resonator has 500 μm radius

The parabolic MEGO reflector has been designed to reflect the collimated beam at a single focal point for selective frequencies where a detector can be placed. To ascertain the unique advantages of this MEGO design, it is instructive to visualize how one would realize such a function using conventional setup shown here for CW THz spectrometer. See Fig. 7a, note the use of multiple parabolic mirrors, one to collimate the beam and another to focus the beam onto a receiver. The new, simpler and more compact set up with MEGO device is shown in Fig. 7b, the difference of the previous reflection set up with the new set up is the lack of second mirror where MEGO device does the converging of the beam as well. We fabricated the device (shown in Fig. 7d) and measured the reflection spectrum of MEGO device showing in Fig. 7c. The reflection spectrum shows a resonant frequency in 91 GHz matching with the simulation results. There is broadening in experimental result at resonant frequency compared to the simulation. The broadening is caused by dimensional variation of disk resonators due to fabrication tolerances. We analyzed the surface area of disk resonators with ImageJ software, and we noticed a variation of 450–600 μm in the radius of the disk resonators caused by variation in the fabrication process. The original simulation had included the reflection spectrum with only a single size of 500 μm of radius for disk resonators ignoring any variation. To accurately map the real-life results, we added some variability in the dimensions of the resonators from 490 to 510 μm. As seen in Fig. S6, there is broadening observed comparing to the simulation of the structure with radius of only 500 μm. Multiple ripples for higher values of reflection are caused by multiple reflections within the sample due to Fabry-Perot interference fringes^62^.

The fabrication approaches proposed promises a consolidation of metamaterial and optical elements with potential for reducing size, weight and complexity of instruments. For example, one could reduce the size of spectrometer for material analysis for on-the-field applications by consolidating several mirrors in the optical path. One can also envision combining different metamaterial designs onto different concave or convex lenses, or embedding a metamaterial within them to realize some unique functionality. This will be basis of future work.

## Discussion

This paper proposes realization of unique microwave and optical devices defined here as MEGO for Metamaterial Embedded Geometrical Optics through a combination of 3D printing, metal deposition and wet etching. We show that we are able to structure the underlying dielectric layers in addition to patterning of the metal layers. As an example, mushroom-type 3D metamaterial absorbers were designed and demonstrated at microwave frequencies. We were able to design and implement MEGO devices with unique functionality, one that takes advantage of the large area 3D printing of dielectrics with the embedding of metal patterns. We show for the first time an omni-directional hemispherical moth-eye absorber except that it is made frequency selective. We also show that we can fuse multiple electromagnetic functions, which traditionally were achieved by using different optical components into a single MEGO (Metamaterial Embedded Geometric Optics) device. We consolidated optical parabolic reflectors with frequency selective transmissive filter operating at 91 GHz into a single device. The functions and utilities of the MEGO devices bring a new toolkit to microwave and optical designers using conventional 3D printers.

## Supplementary Information

Supplementary

## References

[1] Ngo TD, Kashani A, Imbalzano G, Nguyen KTQ, Hui D (2018). Additive manufacturing (3D printing): a review of materials, methods, applications and challenges. Compos. Part B Eng..

[2] Elsallal MW, Hood J, Mcmichael I, Busbee T (2016). 3D printed material characterization for complex phased arrays and metamaterials. Microw. J..

[3] Formlabs Incorporated website. https://formlabs.com/. Accessed on 05/01/2018.

[4] Nanoscribe GmbH website. https://www.nanoscribe.de/. Accessed on 05/01/2018.

[5] Mao M (2017). The emerging frontiers and applications of high-resolution 3D printing. Micromachines.

[6] Mannoor MS (2013). 3D printed bionic ears. Nano Lett..

[7] Symes MD (2012). Integrated 3D-printed reactionware for chemical synthesis and analysis. Nat. Chem..

[8] Jones N (2012). Science in three dimensions: the print revolution. Nature.

[9] Reiffel AJ (2013). High-fidelity tissue engineering of patient-specific auricles for reconstruction of pediatric microtia and other auricular deformities. PLoS One.

[10] Villar G, Graham AD, Bayley H (2013). A tissue-like printed material. Science.

[11] Yeong WY, Chua CK, Leong KF, Chandrasekaran M (2004). Rapid prototyping in tissue engineering: challenges and potential. Trends Biotechnol..

[12] Van Noort R (2012). The future of dental devices is digital. Dent. Mater..

[13] Klein GT, Lu Y, Wang MY (2013). 3D printing and neurosurgery—ready for prime time?. World Neurosurg..

[14] Rad ZF (2017). High-fidelity replication of thermoplastic microneedles with open microfluidic channels. Nat. Publ. Gr..

[15] Nejad HR, Sadeqi A, Kiaee G, Sonkusale S (2018). Low-cost and cleanroom-free fabrication of microneedles. Microsyst. Nanoeng..

[16] Sadeqi, A., Nejad, H. R., Kiaee, G. & Sonkusale, S. Cost-effective fabrication of chitosan microneedles for transdermal drug delivery. In *Proc. International Conference of the IEEE Engineering in Medicine and Biology Society (EMBC)* 5737–5740 (IEEE, Honolulu, HI, 2018). 10.1109/EMBC.2018.8513691 30441639

[17] Valentine AD (2017). Hybrid 3D printing of soft electronics. Adv. Mater..

[18] Chanda D (2011). Large-area flexible 3D optical negative index metamaterial formed by nanotransfer printing. Nat. Nanotechnol..

[19] Bauer J (2017). Nanolattices: an emerging class of mechanical metamaterials. Adv. Mater..

[20] Zheng X (2016). Multiscale metallic metamaterials. Nat. Mater..

[21] Ch EAR (2014). Ultralight, ultrastiff mechanical metamaterials. Science.

[22] Veselago VG (1968). The electrodynamics of substances with simultaneously negative values of ε and μ. Sov. Phys. Uspekhi.

[23] Schurig D (2006). Metamaterial electromagnetic cloak at microwave frequencies. Science.

[24] Rout S, Sonkusale S (2016). Wireless multi-level terahertz amplitude modulator using active metamaterial-based spatial light modulation. Opt. Express.

[25] Xu W, Sonkusale S (2013). Microwave diode switchable metamaterial reflector/absorber. Appl. Phys. Lett..

[26] Chen HT (2009). A metamaterial solid-state terahertz phase modulator. Nat. Photonics.

[27] Melik R, Unal E, Perkgoz NK, Puttlitz C, Demir HV (2009). Metamaterial-based wireless strain sensors. Appl. Phys. Lett..

[28] Ebrahimi A, Withayachumnankul W, Al-Sarawi SF, Abbott D (2014). Metamaterial-inspired rotation sensor with wide dynamic range. IEEE Sens. J..

[29] Ding J (2014). Tuneable complementary metamaterial structures based on graphene for single and multiple transparency windows. Sci. Rep..

[30] Lee SH (2012). Switching terahertz waves with gate-controlled active graphene metamaterials. Nat. Mater..

[31] Hashemi MRM, Yang SH, Wang T, Sepúlveda N, Jarrahi M (2016). Electronically-controlled beam-steering through vanadium dioxide metasurfaces. Sci. Rep..

[32] Shrekenhamer D (2011). High speed terahertz modulation from metamaterials with embedded high electron mobility transistors. Opt. Express.

[33] Sadeqi A, Nejad HR, Sonkusale S (2017). Low-cost metamaterial-on-paper chemical sensor. Opt. Express.

[34] Salim A, Lim S (2018). Review of recent metamaterial microfluidic sensors. Sensors.

[35] Salim A, Memon MU, Lim S (2018). Simultaneous detection of two chemicals using a TE20-mode substrate-integrated waveguide resonator. Sensors.

[36] Srivastava YK, Cong L, Singh R (2017). Dual-surface flexible THz Fano metasensor. Appl. Phys. Lett..

[37] Dong W (2018). Tunable mid-infrared phase-change metasurface. Adv. Opt. Mater..

[38] Wang, B. et al. Metamaterial absorber for THz polarimetric sensing. In *Proc. SPIE Terahertz, RF, Millimeter, and Submillimeter-Wave Technology and Applications*. Vol. 101531 (International Society for Optics and Photonics, San Francisco, CA, 2018).

[39] Sakellari I (2017). 3D chiral plasmonic metamaterials fabricated by direct laser writing: the twisted omega particle. Adv. Opt. Mater..

[40] Marichy C, Muller N, Froufe-Pérez LS, Scheffold F (2016). High-quality photonic crystals with a nearly complete band gap obtained by direct inversion of woodpile templates with titanium dioxide. Sci. Rep..

[41] Muller N, Haberko J, Marichy C, Scheffold F (2014). Silicon hyperuniform disordered photonic materials with a pronounced gap in the shortwave infrared. Adv. Opt. Mater..

[42] Franklin D (2015). Polarization-independent actively tunable colour generation on imprinted plasmonic surfaces. Nat. Commun..

[43] Haberko J, Scheffold F (2012). Fabrication of mesoscale polymeric templates for three-dimensional disordered photonic materials. Opt. Express.

[44] Phillips DB (2012). An optically actuated surface scanning probe. Opt. Express.

[45] Gansel JK (2012). Tapered gold-helix metamaterials as improved circular polarizers. Appl. Phys. Lett..

[46] Park J, Fujita H, Kim B (2011). Fabrication of metallic microstructure on curved substrate by optical soft lithography and copper electroplating. Sens. Actuators, A Phys..

[47] Tao H (2008). Terahertz metamaterials on free-standing highly-flexible polyimide substrates. J. Phys. D. Appl. Phys..

[48] Singh PK, Korolev KA, Afsar MN, Sonkusale S (2011). Single and dual band 77/95/110 GHz metamaterial absorbers on flexible polyimide substrate. Appl. Phys. Lett..

[49] Walia S (2015). Flexible metasurfaces and metamaterials: a review of materials and fabrication processes at micro- and nano-scales. Appl. Phys. Rev..

[50] Yahiaoui R (2015). Multispectral terahertz sensing with highly flexible ultrathin metamaterial absorber. J. Appl. Phys..

[51] Cong L, Srivastava YK, Solanki A, Sum TC, Singh R (2017). Perovskite as a platform for active flexible metaphotonic devices. ACS Photonics.

[52] Kaipa, C. S. R., Yakovlev, A. B. & Silveirinha, M. G. Characterization of negative refraction with multilayered mushroom-type metamaterials at microwaves. *J. Appl. Phys*. **109**, 044901 (2011).

[53] Fernandes, D. E. & Silveirinha, M. G. Bistability in mushroom-type metamaterials. *J. Appl. Phys*. **122**, 014303 (2017).

[54] Sievenpiper D, Zhang L, Jimenez Broas RF, Alexöpolous NG, Yablonovitch E (1999). High-impedance electromagnetic surfaces with a forbidden frequency band. IEEE Trans. Microw. Theory Tech..

[55] Padooru YR (2012). New absorbing boundary conditions and analytical model for multilayered mushroom-type metamaterials: applications to wideband absorbers. IEEE Trans. Antennas Propag..

[56] Tretyakov SA, Maslovski SI (2003). Thin absorbing structure for all incidence angles based on the use of a high-impedance surface. Microw. Opt. Technol. Lett..

[57] Solidworks software website. http://www.solidworks.com/. Accessed on 05/01/2018.

[58] Toptica Photonics website. http://www.toptica.com/. Accessed on 05/01/2018.

[59] Rout S, Sonkusale SR (2016). A low-voltage high-speed terahertz spatial light modulator using active metamaterial. APL Photonics.

[60] Wang J, Liu S, Guruswamy S, Nahata A (2014). Injection molding of free‐standing, three‐dimensional, all‐metal terahertz metamaterials. Adv. Opt. Mater..

[61] AutoCAD software website. https://www.autodesk.com/. Accessed on 05/01/2018.

[62] Roggenbuck A (2010). Coherent broadband continuous-wave terahertz spectroscopy on solid-state samples. New J. Phys..

