# Supplementary material for: Three dimensional printing of metamaterial embedded geometrical optics (MEGO)
Source: Microsyst Nanoeng. 2019 Apr 8;5:16. doi: 10.1038/s41378-019-0053-6 (PMC6451962; doi:10.1038/s41378-019-0053-6)
Supplement: Supplementary file 1 — Supplementary [file 41378_2019_53_MOESM1_ESM.docx]

**Supplementary file**

Three dimensional printing of metamaterial embedded geometrical optics (MEGO)

Aydin Sadeqi^*^, Hojatollah Rezaei Nejad^*^, Rachel E. Owyeung and Sameer Sonkusale

Microsystems & Nanoengineering (2018)

Nano Lab, Department of Electrical and Computer Engineering, Tufts University, Medford, MA 02155, USA

^*^These authors contributed equally to this work.

**Supplementary Materials and Methods**


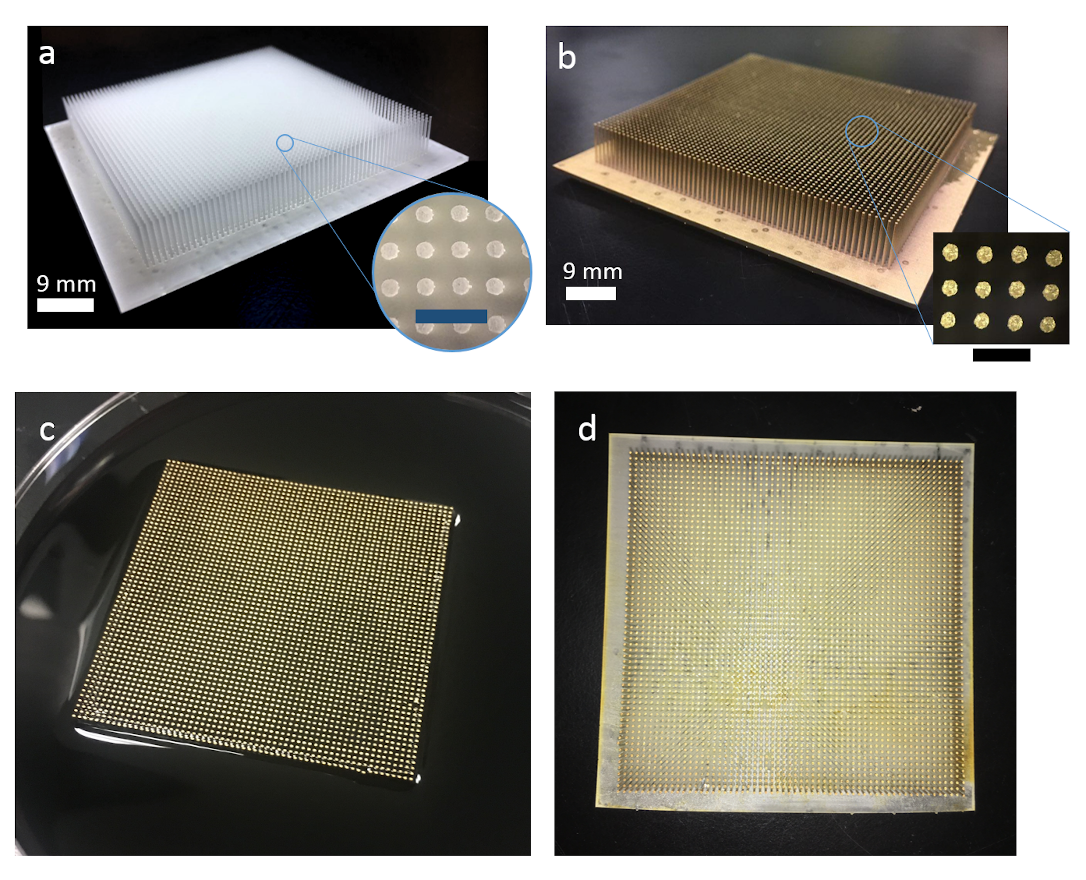


**Fig. S1** (a) 3D printed device (b) Sputtered device (c) Etching of the unwanted areas (pedestals and substrate) (d) final SRR MEGO device.


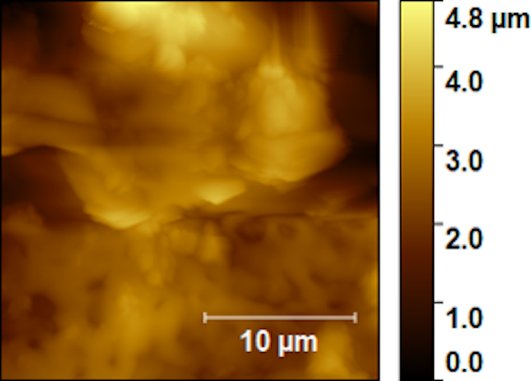


**Fig. S2** Atomic Force Microscopy from the surface of the resonators


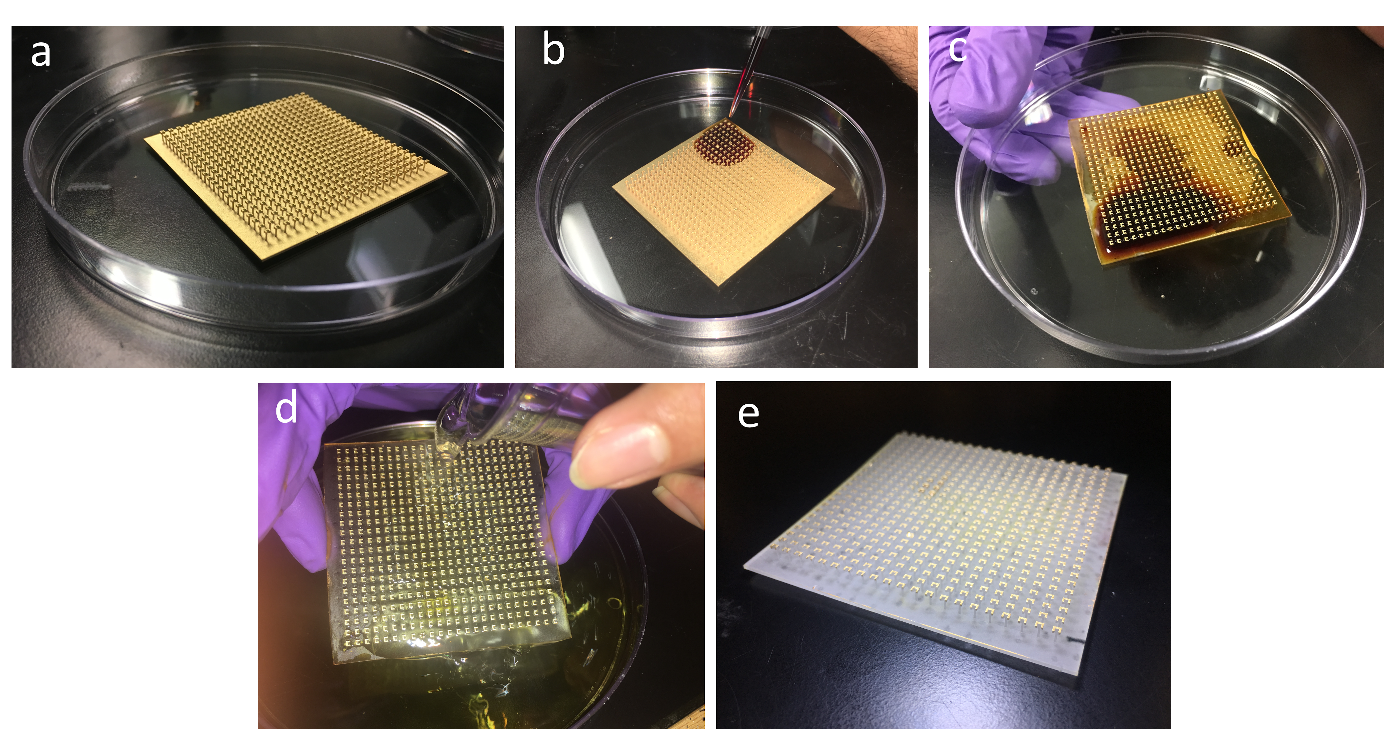


**Fig. S3** (a) Sputtered SRR Mushroom MEGO device (b,c) Etching of the unwanted areas (pedestals and substrate) (d) Rinsing the device (e) final SRR Mushroom MEGO device.


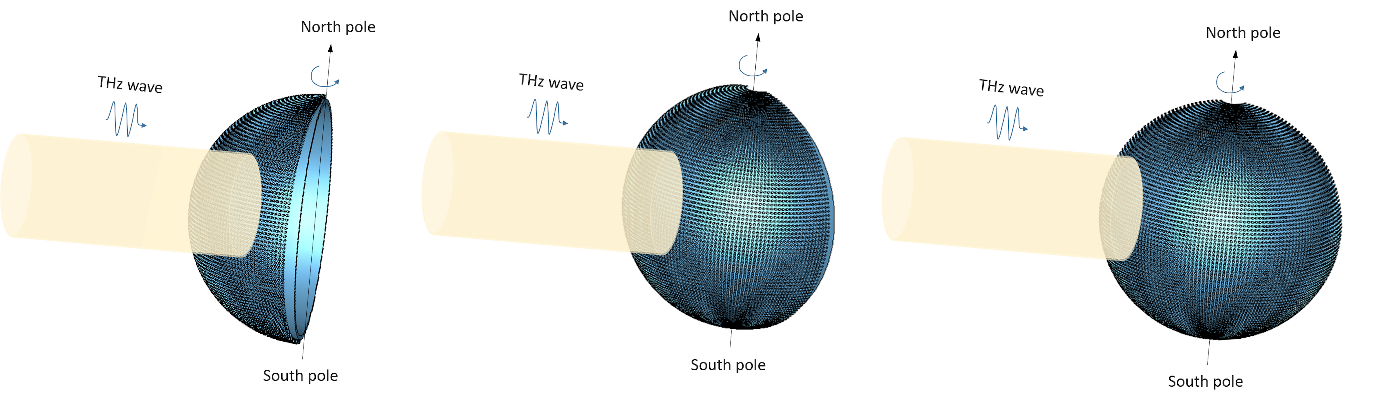


**Fig. S4** Different measurement angles for moth-eye absorber.


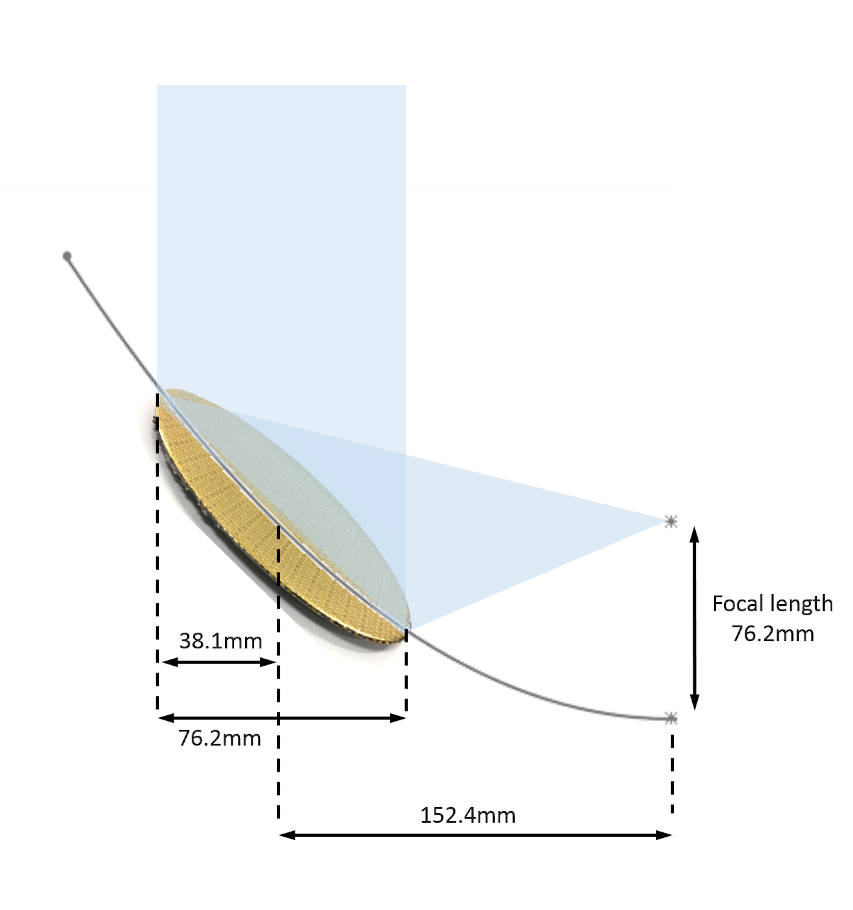


**Fig. S5** Parabolic reflector dimensions.


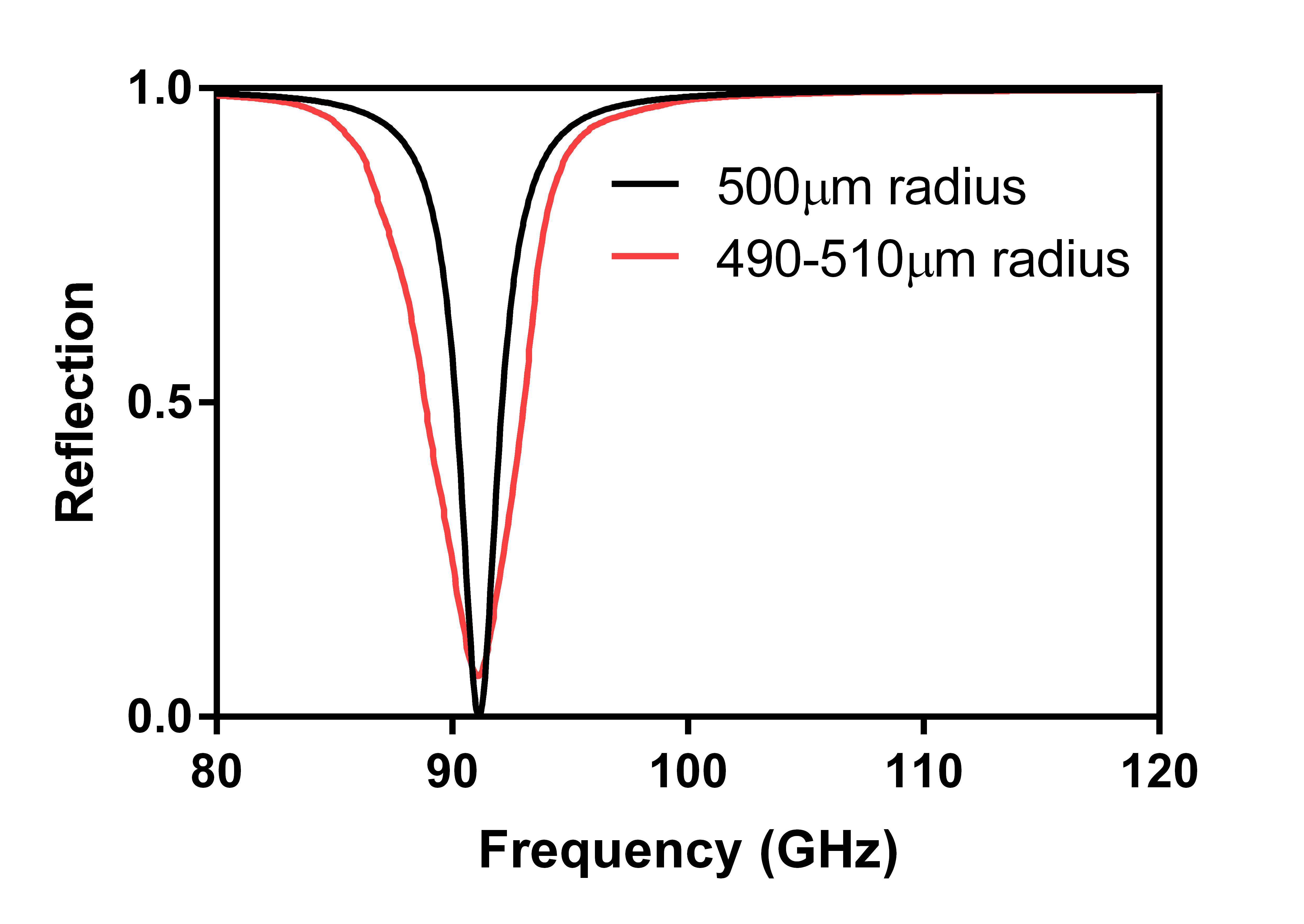


**Fig. S6** Comparing the simulation result for disk resonators with radius of 500μm and for resonators with variation between 490μm ~ 510μm shows broadening of resonance as seen in experimental data.
